# An In Vitro and In Vivo Assessment of Acidogenicity of Several Alcoholic and Non‐Alcoholic Beverages

**DOI:** 10.1002/cre2.70462

**Published:** 2026-10-04

**Authors:** Riccardo Guazzo, Eriberto Bressan, Anna Lehrkinder, Guglielmo Campus, Edoardo Stellini, Cristiano Tomasi, Peter Lingström

**Affiliations:** ^1^ Department of Neurosciences, Institute of Clinical Dentistry University of Padua Padua Italy; ^2^ Department of Cariology, Institute of Odontology, Sahlgrenska Academy University of Gothenburg Gothenburg Sweden; ^3^ Department of Periodontology, Institute of Odontology, Sahlgrenska Academy University of Gothenburg Gothenburg Sweden

**Keywords:** acidogenicity, erosion, pH, sparkling wine, wine

## Abstract

**Objectives:**

This study evaluated the acidogenic potential of selected alcoholic and non‐alcoholic beverages using complementary in vitro (initial pH and titratable acidity) and in vivo (tooth‐surface pH after exposure) methods.

**Materials and Methods:**

Two sparkling wines (traditional and tank method), red wine, white wine, an alcoholic soft drink, a non‐alcoholic soft drink, pilsner beer, and orange juice were examined. In vitro, initial pH and titratable acidity were measured at room temperature (22°C). In vivo, 19 healthy adults held 10 mL of each selected beverage intraorally for 1 min, after which tooth‐surface pH was recorded at predefined time points for up to 30 min using a microelectrode. Data were analyzed using ANOVA with post‐hoc Tukey tests (*p* < 0.05).

**Results:**

In vitro, the non‐alcoholic soft drink had the lowest initial pH and beer the highest. Orange juice and sparkling wine produced by the traditional method showed the greatest titratable acidity. All beverages except water caused an immediate in vivo pH decrease, followed by recovery toward baseline within 10–15 min. The lowest mean tooth‐surface pH was 6.2 for the traditional‐method sparkling wine.

**Conclusions:**

Alcoholic and non‐alcoholic beverages differed substantially in their acidogenic properties. Salivary buffering promoted rapid pH recovery, but beverages with high titratable acidity, particularly traditional‐method sparkling wine and orange juice, produced a more sustained acid challenge. Because this study measured short‐term pH responses rather than dental hard‐tissue loss, the findings should be interpreted as indicators of acidogenicity rather than direct measures of erosive damage.

## 1 Introduction

Erosive tooth wear (ETW) is a multifactorial condition characterized by irreversible loss of mineralized tooth structure, with dietary acids representing one of the most relevant extrinsic etiological factors (Schlueter et al. 2020; Dallavilla et al. 2024; Schiffner et al. 2025). Numerous epidemiological studies have reported increasing consumption of acidic beverages—such as soft drinks, fruit juices, and alcoholic drinks—along with parallel increases in the prevalence of erosive lesions among children, adolescents, and adults (Cavadini 2000; Johansson et al. 2002; Dugmore and Rock 2004; Schlueter et al. 2020; Cortese et al. 2024; Dallavilla et al. 2024; Schiffner et al. 2025; Rolim de Lavôr et al. 2025). The erosive potential of a beverage depends on several physicochemical factors, including initial pH, titratable acidity, type and concentration of organic acids, calcium and phosphate content, and degree of saturation with respect to hydroxyapatite (Larsen and Pearce 1997; Dugmore and Rock 2004; Barbour et al. 2011; Jager et al. 2012; Tenuta et al. 2015). The acidity of wine on the permanent dentition can be quite detrimental, with an increased risk of erosion of dental hard tissue (Barbour et al. 2011; Tenuta et al. 2015). Global consumption of alcohol‐containing products has increased since 2010 and is forecast to reach US$ 447.1 billion in 2025 (Johansson et al. 2014; Melbye et al. 2020). The sparkling wine market was valued at USD 290.1 billion in 2024 and is forecast to grow by steady per capita consumption of sparkling wine across the globe (Advisory Vine Industry Network 2024; Statista 2024). Among acidic drinks, wine has received particular attention due to its low pH and its widespread consumption. Sparkling wines may be of special interest because of their higher acidity and carbonation, which could enhance their acidogenic potential. However, current evidence is largely based on in vitro studies that do not fully simulate the complex protective role of saliva. In vivo, several host‐related factors—including salivary flow rate, buffer capacity, pellicle formation, and oral clearance—substantially modulate the impact of dietary acids (Chikte et al. 2003; Lan et al. 2023; Barac et al. 2023; Lupi‐Pegurieret al. 2003; Enax et al. 2024; Madariaga et al. 2025; Nobre et al. 2025). Saliva can counteract pH drops through dilution, buffering, and remineralization, meaning that beverage acidity alone does not directly predict actual intraoral acidogenicity or erosion risk. Real‐time intraoral pH measurements therefore provide important complementary information beyond laboratory assays.

Despite this, in vivo studies investigating short‐term intraoral pH changes after consumption of alcoholic beverages remain limited (Lupi‐Pegurier et al. 2003; Barac et al. 2023; Lan et al. 2023). Furthermore, most available studies do not assess beverage acidity and the subsequent intraoral pH response within the same experimental framework. Therefore, the aim of this study was to assess and compare the acidogenic potential of selected alcoholic and non‐alcoholic beverages using a combined in vitro and in vivo approach. Specifically, we evaluated initial beverage pH and titratable acidity under laboratory conditions and investigated the magnitude and duration of tooth‐surface pH changes following standardized intraoral exposure.

## 2 Materials and Methods

### 2.1 Beverages

Eight commercially available beverages were selected to represent a range of alcoholic and non‐alcoholic drinks frequently consumed by the general population. The products included two sparkling wines (one produced by the traditional method and one by the tank method), a red wine, a white wine, an alcoholic soft drink, a non‐alcoholic soft drink, a pilsner beer, and orange juice. All beverages were tested at room temperature (22°C) for methodological standardization.

### 2.2 In Vitro Investigation

Beverages were stored at room temperature for 60 min before testing. For initial pH measurement, 80 mL of each beverage was placed in a beaker and measured with a calibrated pH electrode (Orion 9102BNWP, Thermo Scientific, USA) connected to an Orion 720 A analyzer (Orion Research Inc., USA). The electrode was calibrated immediately before measurement using standard pH 4.0 and 7.0 buffers. Each beverage was stirred until a stable reading was obtained, and three independent measurements were recorded. Mean values and standard deviations were calculated (Owens 2007).

Titratable acidity was assessed by titrating 40 mL of each beverage with 0.1 M sodium hydroxide (NaOH; Merck KGaA, Darmstadt, Germany). Using continuous stirring, NaOH was added in 0.5 mL (2 mg NaOH) increments. The volume required to reach pH 5.0, 6.0, 7.0, and 8.3 was recorded. All titrations were performed in triplicate. Values are reported as mg NaOH required to reach the target pH.

### 2.3 In Vivo Investigation

The study was conducted in accordance with the Declaration of Helsinki and was approved by the Ethics Committee of the University of Padua (protocol 0017061). Written informed consent was obtained from all participants. Twenty healthy volunteers (10 men and 10 women) from the Faculty of Odontology, University of Padua, were recruited; one female participant discontinued because of pregnancy, leaving 19 participants who completed the study. Inclusion criteria were age ≥ 18 years, full permanent dentition without fixed or removable prostheses, good general health, and normal baseline salivary function. Exclusion criteria were gastroesophageal reflux disease (GERD), enamel defects or developmental anomalies, xerostomia or medication‐induced hyposalivation, use of medications affecting salivary flow, smoking, pregnancy, high plaque scores, a history of substantial erosive tooth wear, and active caries. The study was exploratory. A target sample of approximately 20 participants was selected on the basis of previous studies using comparable microelectrode‐based intraoral pH protocols; under an assumed SD of approximately 0.4 pH units, this sample size was considered adequate to detect differences of approximately 0.3–0.4 pH units with about 80% power at *α* = 0.05.

Participants refrained from eating or drinking, except water, for 2 h before testing. The in vivo test beverages were the two sparkling wines, the alcoholic and non‐alcoholic soft drinks, beer, and orange juice; still water served as the control. The order of beverages was randomized using Microsoft Excel for Mac (Version 16.102.2). Each participant held 10 mL of the assigned beverage intraorally for 1 min before expectoration. This standardized exposure was selected to facilitate comparison between beverages, although it does not reproduce all real‐life drinking patterns (Johansson et al. 2014).

Stimulated saliva flow rate, buffer capacity (Saliva‐Check; Nimi Chemical Industrial Co. Ltd., Japan), and DFT index were assessed before the study.

Tooth‐surface pH was measured at baseline and at 1, 2, 3, 5, 7, 10, 15, 20, and 30 min post‐exposure. Measurements were obtained using a microelectrode pH sensor (AMANI‐650; 0.65 mm tip, 20 mm length) connected to a pH meter with mV display (Hanna Instruments Italia) by the micro‐touch method (Lingström et al. 1993; Johansson et al. 2014; Cocco et al. 2017). The microelectrode was calibrated using pH 4.0 and 7.0 buffer solutions before each series of measurements. To minimize drift, calibration was checked periodically throughout the session and recalibrated if deviation exceeded 0.02 pH units.

The microelectrode was placed in the same area of the tooth in all subjects and at all time points. Measurements were performed at three intraoral sites: (1) the vestibular surface of the upper right central incisor, (2) the palatal surface of the upper right central incisor, and (3) the top of the mesio‐buccal cusp tip of the first upper right molar (Figure 1).

**Figure 1:**
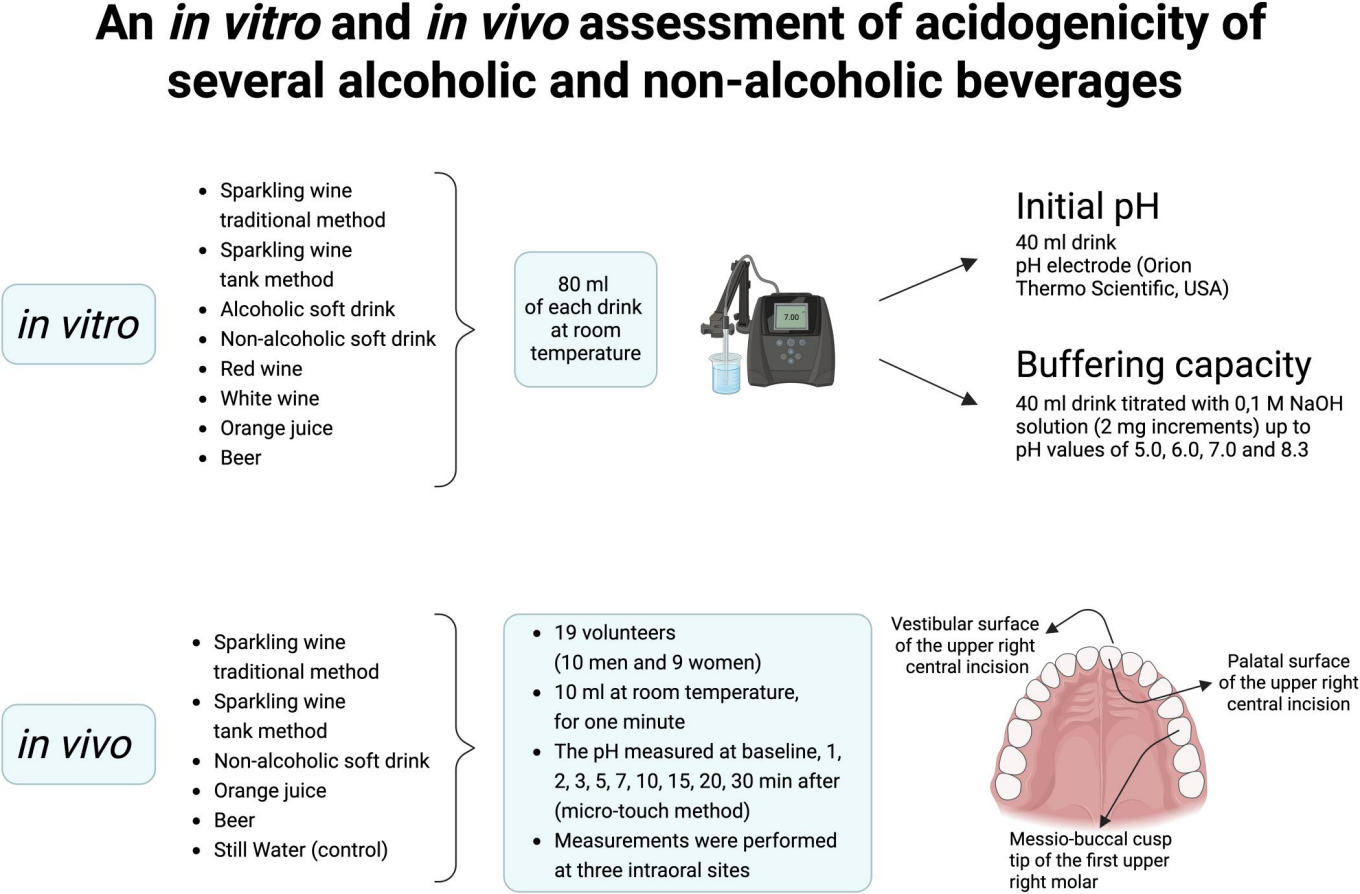
Flow chart of the study design and methods.

### 2.4 Statistical Analysis

Descriptive statistics (mean, SD, minimum, and maximum) were computed. For the in vivo pH‐time curves, AUC7.5 was calculated as the integrated difference between the operational reference pH of 7.5 and the observed tooth‐surface pH over time. The value 7.5 was selected solely as a common upper reference because all recorded measurements were below this level; it was not intended to represent a biological critical pH. Thus, larger AUC7.5 values indicate a greater and/or more prolonged acidogenic response. Beverage comparisons were performed using one‐way ANOVA followed by Tukey post‐hoc tests, with statistical significance set at *p* < 0.05. For in vitro pH and titration measurements, two‐way ANOVA was additionally applied. Normality and homogeneity of variances were assessed before ANOVA.

## 3 Results

### 3.1 In Vitro Investigation

Initial pH values and the amount of NaOH required to reach the target pH levels are summarized in Table 1. Significant differences were observed across beverages for both initial pH (ANOVA, *p* = 0.01) and titratable acidity (*p* < 0.01). Beer had the highest initial pH (4.43 ± 0.01), whereas the non‐alcoholic soft drink had the lowest (2.56 ± 0.10). The amount of NaOH required to reach pH 8.3 varied substantially among beverages. Orange juice (195.33 ± 4.83 mg) and sparkling wine produced by the traditional method (193.33 ± 13.07 mg) required the greatest amounts, whereas beer required the least (66.00 ± 4.00 mg) (Figure 2). Post‐hoc comparisons were statistically significant for most beverage pairs. Non‐significant comparisons were alcoholic soft drink versus orange juice (*p* = 0.62), white wine versus alcoholic soft drink (*p* = 0.14), non‐alcoholic soft drink versus beer (*p* = 0.09), and orange juice versus traditional‐method sparkling wine (*p* = 0.48).

**Table 1:** In vitro measurements of pH, NaOH.

	Initial pH (MV ± SD)	NaOH (mg) (MV ± SD)
Sparkling wine traditional method	2.82 ± 0.03	193.33 ± 13.07
Sparkling wine tank method	2.98 ± 0.02	152.67 ± 5.03
Red wine	3.41 ± 0.03	132.67 ± 4.16
White wine	3.21 ± 0.01	178.67 ± 8.08
Alcoholic soft drink	2.94 ± 0.02	190.67 ± 11.72
Non‐alcoholic soft drink	2.56 ± 0.10	78.68 ± 5.03
Beer	4.43 ± 0.01	66.00 ± 4.00
Orange juice	3.56 ± 0.01	195.33 ± 4.83
*ANOVA one way p‐value*	0.01	*< *0.01

*Note:* NaOH mg values represent the quantity required to reach pH 8.3. Differences assessed by one‐way ANOVA.

**Figure 2:**
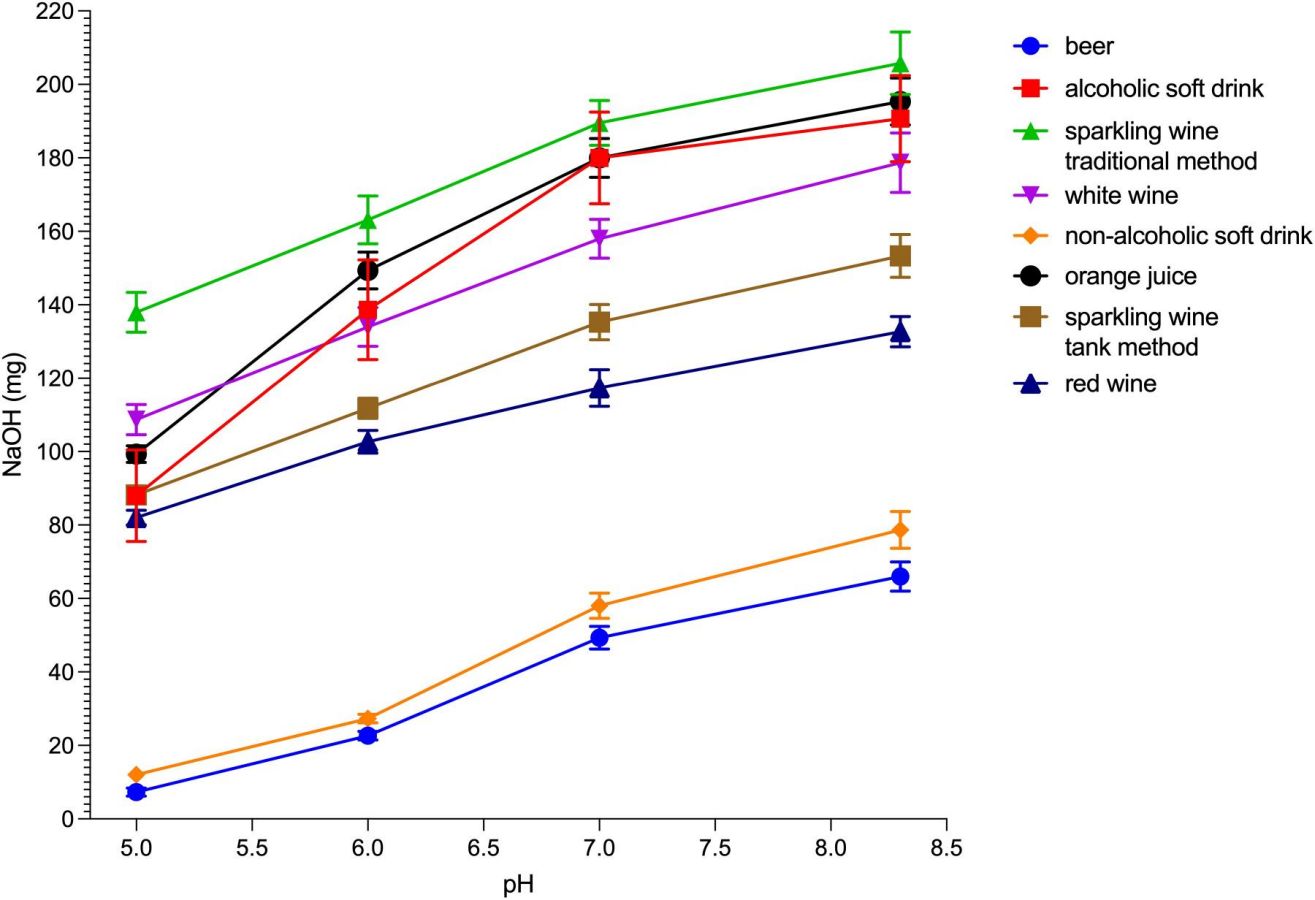
In vitro titration profiles of the tested beverages, illustrating changes in pH as increasing amounts of NaOH were added during neutralization.

### 3.2 In Vivo Investigation

Demographic and baseline clinical characteristics of the participants are presented in Table 2. One female participant discontinued due to pregnancy; 19 individuals completed all measurements. Figure 3 illustrates the pH curves across the 30‐min period following intraoral exposure. All beverages except still water caused an immediate reduction in tooth‐surface pH. Recovery toward baseline occurred within 10–15 min for all test products.

**Table 2:** In vivo study. Vital statistics and oral conditions of the volunteers (*n* = 19).

Age (years)	Mean ± SD Range (min–max)	32.3 ± 9.3 24–57
Sex	Female	9
	Male	10
Saliva buffer capacity (pH)	High	19
Stimulated saliva secretion rate (mL/min)	Mean ± SD	10.9 ± 5.2
DFT	Mean ± SD	3.1 ± 2.7

**Figure 3:**
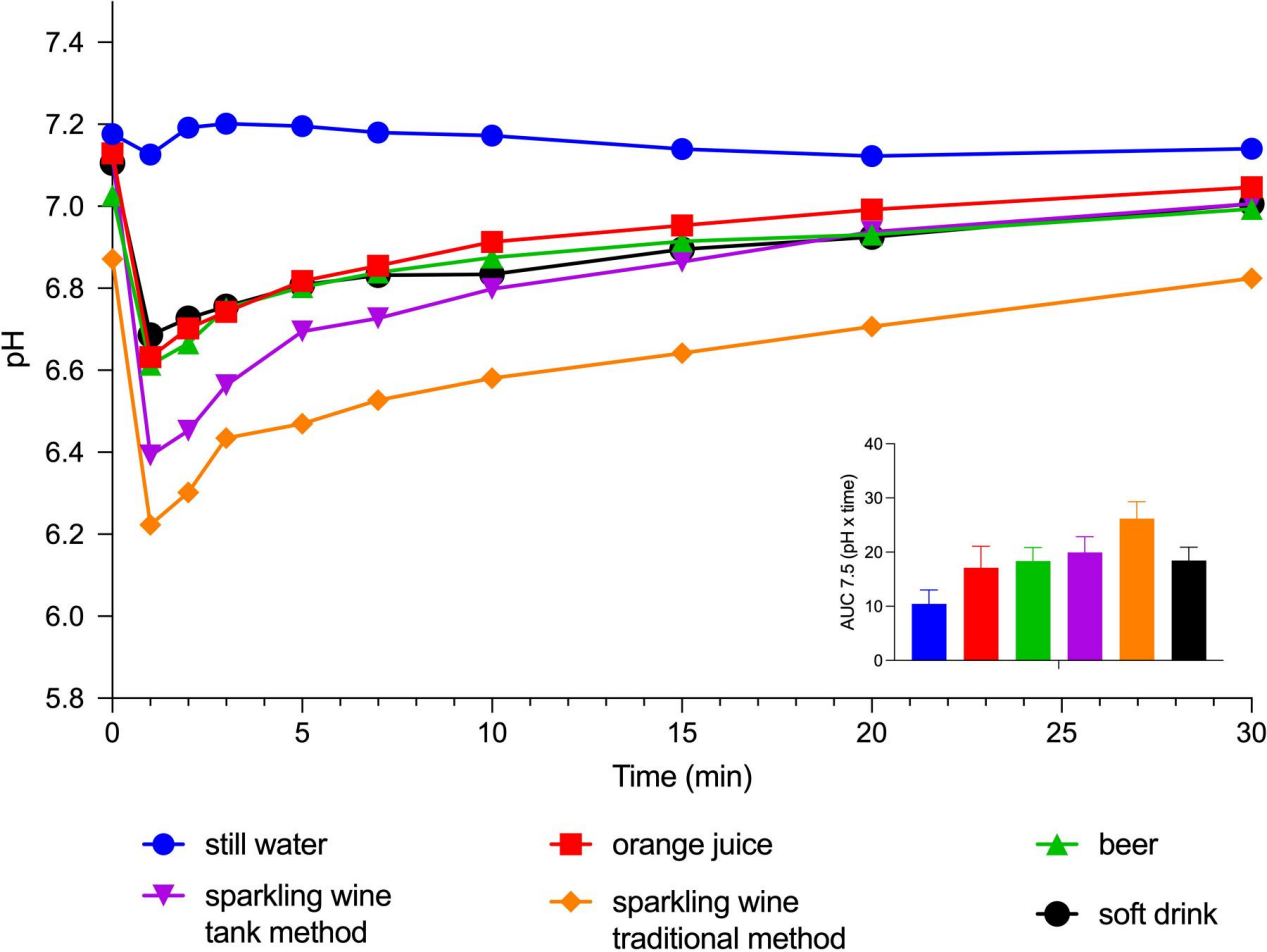
In vivo tooth‐surface pH curves over 30 min following exposure to each beverage. AUC7.5 values summarize the magnitude and duration of the acidogenic response relative to the operational reference pH of 7.5.

Minimum pH values, maximum pH fall, final pH values, and AUC7.5 results are presented in Table 3. Minimum pH differed significantly among beverages (*p* = 0.04). Traditional‐method sparkling wine reached the lowest mean value (6.20 ± 0.40). Maximum pH fall also differed significantly among beverages (*p* = 0.03): the traditional‐ and tank‐method sparkling wines produced the greatest decreases (0.80 ± 0.30 and 0.80 ± 0.40, respectively), whereas the non‐alcoholic soft drink and beer produced smaller decreases (0.50 ± 0.20 for both).

**Table 3:** In vivo pH values measured at the tooth surface following 1‐min intraoral exposure to each beverage.

	Sparkling wine traditional method (Mean ± SD)	Sparkling wine tank method (Mean ± SD)	Non‐alcoholic soft drink (Mean ± SD)	Orange juice Mean ± SD	Beer (Mean ± SD)	*p* value	Still water (control) (Mean ± SD)
Baseline pH	6.9 ± 0.3	7.1 ± 0.2	7.1 ± 0.2	7.1 ± 0.3	7.0 ± 0.2	N.S.	7.2 ± 0.3
Min pH	6.2 ± 0.4	6.3 ± 0.4	6.6 ± 0.3	6.6 ± 0.4	6.6 ± 0.3	0.04	7.1 ± 0.3
Max pH‐fall	0.8 ± 0.3	0.8 ± 0.4	0.5 ± 0.2	0.6 ± 0.4	0.5 ± 0.2	0.03	0.2 ± 0.1
Final pH	6.8 ± 0.3	7.0 ± 0.3	7.0 ± 0.2	7.0 ± 0.3	6.9 ± 0.2	N.S.	7.1 ± 0.3
AUC7.5	26.2 ± 3.1	19.9 ± 2.9	18.4 ± 2.4	17.1 ± 3.9	18.4 ± 2.5	0.02	10.4 ± 2.6

*Note:* Parameters include baseline pH, minimum pH, maximum pH fall, final pH, and AUC7.5. Statistical differences were assessed using one‐way ANOVA and Tukey post‐hoc tests.

Final pH values did not differ significantly from baseline for any beverage (*p* > 0.05). AUC7.5 differed among beverages and water (ANOVA, *p* = 0.02). The highest AUC7.5 was recorded for traditional‐method sparkling wine (26.2 ± 3.1). Orange juice (17.1 ± 3.9), beer (18.4 ± 2.5), and the non‐alcoholic soft drink (18.4 ± 2.4) showed lower values, while still water had the lowest AUC7.5 (10.4 ± 2.6).

## 4 Discussion

Erosive tooth wear is a cumulative chemical‐mechanical process in which non‐bacterial acids contribute to irreversible loss of dental hard tissue (Schlueter et al. 2020). For dietary exposures, erosive potential cannot be inferred from beverage pH alone; titratable acidity, acid type and concentration, mineral composition, degree of saturation with respect to dental apatite, exposure time, and host factors all influence the biological response (Larsen and Pearce 1997; Barbour et al. 2011; Jager et al. 2012; Tenuta et al. 2015). Accordingly, the present study combined laboratory characterization of beverage acidity with direct measurement of the short‐term intraoral pH response.

The principal finding was that the ranking of beverages by initial pH did not fully parallel their behavior during neutralization or after intraoral exposure. The non‐alcoholic soft drink had the lowest initial pH, whereas orange juice and traditional‐method sparkling wine required the greatest amount of NaOH for neutralization. This distinction is clinically relevant because initial pH reflects the immediate hydrogen‐ion activity, while titratable acidity represents the quantity of base required to raise pH and therefore provides information on how long an acidic challenge may persist. Tenuta et al. (2015), in an in vivo crossover study, similarly showed that beverage titratable acidity influenced the time required for salivary pH recovery more strongly than initial beverage pH. Together with earlier physicochemical studies (Larsen and Pearce 1997; Barbour et al. 2011), our results support considering both parameters when characterizing acidogenic beverages.

The comparatively high acidity of wine observed here is consistent with previous experimental evidence. Wine exposure has been associated with enamel softening, reduced microhardness, and changes in surface morphology in vitro (Chikte et al. 2003; Lupi‐Pegurier et al. 2003; Barac et al. 2023; Lan et al. 2023). Wine‐specific effects are not determined by pH alone: organic acids, mineral content, and interactions with the acquired salivary pellicle may modify erosion kinetics (Barbour et al. 2011; Carvalho et al. 2021). The traditional‐method sparkling wine produced both a large neutralization requirement in vitro and the greatest in vivo AUC7.5, suggesting that its acid load was comparatively persistent under the standardized conditions of this study. However, AUC7.5 should be interpreted as a comparative descriptor of the measured pH response rather than as an established biological threshold for erosion.

In vivo, all tested beverages except water caused an immediate decrease in tooth‐surface pH, followed by recovery toward baseline within approximately 10–15 min. This pattern is biologically plausible because saliva dilutes and clears acids, provides bicarbonate and other buffering systems, contributes calcium and phosphate for remineralization, and supports formation of the acquired pellicle (Johansson et al. 2002; Carvalho et al. 2021; Enax et al. 2024; Nobre et al. 2025). The relatively rapid recovery observed in the present cohort should therefore be interpreted in the context of the participants’ favorable salivary status: all 19 participants had high buffer capacity (Table 2). Previous microelectrode studies have likewise shown that intraoral pH responses depend on both beverage‐related and individual factors, including drinking method and oral clearance (Lingström et al. 1993; Johansson et al. 2014; Cocco et al. 2017).

Traditional‐method sparkling wine reached the lowest mean tooth‐surface pH (6.20 ± 0.40) and produced the largest AUC7.5. The dispersion around the mean indicates appreciable interindividual variability, but the present dataset does not support assigning a single universal “critical pH” to each participant. Critical pH depends on local mineral saturation and differs between enamel and dentine; it is therefore preferable to interpret the measured pH values as indicators of acid challenge rather than direct evidence of tissue demineralization (Larsen and Pearce 1997; Barbour et al. 2011). Beer, by contrast, showed the highest initial pH and the lowest titratable acidity in vitro, which is consistent with its comparatively modest in vivo pH response. The orange juice result further illustrates that a beverage with a higher initial pH can nevertheless impose a substantial neutralization demand because of high titratable acidity (Tenuta et al. 2015).

The difference between the in vitro and in vivo observations also emphasizes the limitations of laboratory erosion models. In vitro studies can demonstrate enamel surface softening or morphological change after exposure to alcoholic and acidic beverages (Chikte et al. 2003; Lupi‐Pegurier et al. 2003; Barac et al. 2023; Lan et al. 2023), but they cannot fully reproduce salivary clearance, buffering, remineralization, or pellicle‐mediated protection (Carvalho et al. 2021; Enax et al. 2024). Conversely, rapid pH recovery after a single exposure should not be interpreted as absence of long‐term risk. Erosive tooth wear is cumulative, and exposure frequency and duration are important determinants of tissue loss (Jager et al. 2012; Schlueter et al. 2020). Epidemiological and risk‐group data also support greater concern in individuals with frequent acidic beverage exposure or reduced protective host factors (Dallavilla et al. 2024; Rolim de Lavôr et al. 2025).

Several methodological considerations limit the external validity of the present findings. First, beverages were tested at 22°C for standardization, whereas many of these products are usually consumed chilled; temperature may modify sensory stimulation, salivary flow, and clearance. Second, participants held 10 mL intraorally for 1 min, a controlled protocol that facilitates between‐beverage comparison but does not reproduce the variability of normal sipping, swallowing, beverage volume, or drinking duration (Johansson et al. 2014). Third, the study population consisted of healthy adults with high salivary buffer capacity, so the magnitude and duration of pH changes may be greater in individuals with hyposalivation or other risk factors. Finally, the study measured pH dynamics and titratable acidity but did not directly quantify enamel or dentine softening, surface loss, or mineral dissolution. Acidogenicity and erosive tissue loss are related but not interchangeable outcomes (Barbour et al. 2011; Schlueter et al. 2020).

A further analytical limitation is the use of pH 7.5 as the reference for AUC calculation. This value was selected to provide a common upper reference for all observed pH curves and was not intended as an enamel‐ or dentine‐specific critical threshold. AUC7.5 therefore enables internal comparison of the magnitude and duration of acidification among beverages, but it should not be interpreted as a validated clinical erosion‐risk cut‐off.

Within these limitations, the data indicate that a single standardized exposure produces a transient acidogenic challenge in healthy adults and that saliva can restore tooth‐surface pH relatively rapidly. This finding should not be extrapolated to repeated daily exposures or to susceptible individuals. Frequent acidic beverage intake, reduced salivary flow or buffering, gastroesophageal reflux, and occupational exposure to wine are recognized contexts in which cumulative erosive tooth wear may be more likely (Dallavilla et al. 2024; Vieira 2021; Schlueter et al. 2020).

From a clinical perspective, dietary advice should therefore consider not only the nominal pH of a beverage but also exposure frequency, drinking pattern, and patient‐specific protective factors. Strategies that reduce the frequency and contact time of acidic drinks are consistent with current concepts of erosive tooth‐wear prevention (Schlueter et al. 2020; Dallavilla et al. 2024). Future studies should combine intraoral pH monitoring with direct measures of dental hard‐tissue change and include participants with a wider range of salivary characteristics to determine how short‐term acidogenicity translates into cumulative erosive damage.

## 5 Conclusions

This study demonstrated substantial differences in the acidogenic properties of alcoholic and non‐alcoholic beverages using complementary in vitro and in vivo methods. Orange juice and traditional‐method sparkling wine showed the greatest titratable acidity, while traditional‐method sparkling wine produced the largest short‐term in vivo acidogenic response. All tested beverages except water caused an immediate decrease in tooth‐surface pH, followed by recovery toward baseline within approximately 10–15 min.

The findings should be interpreted as measures of short‐term acidogenicity rather than direct evidence of enamel or dentine loss. The relatively rapid recovery observed in this healthy, high‐buffer‐capacity cohort highlights the protective role of saliva, but does not exclude cumulative effects from frequent or prolonged acidic exposures.

Overall, assessment of potential erosive risk should integrate beverage properties, exposure pattern, and host susceptibility. Further studies combining intraoral pH measurements with direct hard‐tissue outcomes are needed to determine the clinical significance of the differences observed here.

## Author Contributions

**Riccardo Guazzo:** conceptualization, visualization, writing – original draft, writing – review and editing. **Anna Lehrkinder:** conceptualization, visualization, writing – original draft, writing – review and editing, funding acquisition, formal analysis. **Guglielmo Campus:** visualization, writing – original draft, writing – review and editing. **Edoardo Stellini:** conceptualization, writing – original draft, writing – review and editing. **Cristiano Tomasi:** conceptualization, data curation, writing – review and editing. **Eriberto Bressan:** conceptualization, data curation, writing – review and editing. **Peter Lingström:** conceptualization, writing – original draft, writing – review and editing.

## Funding

The authors have nothing to report.

## Ethics Statement

The study on human participants was in accordance with local legislation and institutional requirements. The study was approved by the local institutional review board and ethics committee (protocol number 0017061). All procedures performed were in accordance with the ethical standards of the 1964 Helsinki Declaration and its later amendments.

## Conflicts of Interest

The authors declare no conflicts of interest.

## Data Availability

The dataset generated during and/or analyzed during the current study is not publicly available due to data holder restrictions. The data are available on request.
